# Genetic diversity and differentiation in reef-building *Millepora* species, as revealed by cross-species amplification of fifteen novel microsatellite loci

**DOI:** 10.7717/peerj.2936

**Published:** 2017-02-23

**Authors:** Caroline E. Dubé, Serge Planes, Yuxiang Zhou, Véronique Berteaux-Lecellier, Emilie Boissin

**Affiliations:** 1 EPHE, PSL Research University, UPVD, CNRS, USR 3278 CRIOBE, F-66860, Perpignan, France; 2 Laboratoire d’excellence “CORAIL”, EPHE, PSL Research University, UPVD, CNRS, USR 3278 CRIOBE, Papetoai, Moorea, French Polynesia; 3 ENTROPIE, UMR250/9220-IRD/CNRS/UR, Laboratoire d’excellence “CORAIL”, Nouméa, New-Caledonia

**Keywords:** Genetic diversity, Genetic distance, Cross-species transferability, Microsatellites, *Millepora*

## Abstract

Quantifying the genetic diversity in natural populations is crucial to address ecological and evolutionary questions. Despite recent advances in whole-genome sequencing, microsatellite markers have remained one of the most powerful tools for a myriad of population genetic approaches. Here, we used the 454 sequencing technique to develop microsatellite loci in the fire coral *Millepora platyphylla*, an important reef-builder of Indo-Pacific reefs*.* We tested the cross-species amplification of these loci in five other species of the genus *Millepora* and analysed its success in correlation with the genetic distances between species using mitochondrial 16S sequences. We succeeded in discovering fifteen microsatellite loci in our target species *M. platyphylla,* among which twelve were polymorphic with 2–13 alleles and a mean observed heterozygosity of 0.411. Cross-species amplification in the five other *Millepora* species revealed a high probability of amplification success (71%) and polymorphism (59%) of the loci. Our results show no evidence of decreased heterozygosity with increasing genetic distance. However, only one locus enabled measures of genetic diversity in the Caribbean species *M. complanata* due to high proportions of null alleles for most of the microsatellites. This result indicates that our novel markers may only be useful for the Indo-Pacific species of *Millepora.* Measures of genetic diversity revealed significant linkage disequilibrium, moderate levels of observed heterozygosity (0.323–0.496) and heterozygote deficiencies for the Indo-Pacific species. The accessibility to new polymorphic microsatellite markers for hydrozoan *Millepora* species creates new opportunities for future research on processes driving the complexity of their colonisation success on many Indo-Pacific reefs.

## Introduction

Coral reefs are increasingly threatened by chronic and acute stressors (Bellwood et al., 2004) and are expected to be highly vulnerable to future climate change due to rapidly increasing sea surface temperatures and ocean acidification (Hoegh-Guldberg et al., 2007; Pandolfi et al., 2011; Kuffner et al., 2015). These anthropogenic disturbances can further change the biodiversity in coral reefs and may hamper their capacity to deliver important sources of ecosystem services to millions of people (Wilkinson, 2008; Cardinale et al., 2012). The capacity of reef organisms to survive and adapt to such environmental changes will partially depend on their levels of genetic diversity, which is key for a species’ ability to persist in changing environments (Frankham, 2005; Barrett & Schluter, 2008; Hoffmann & Sgrò, 2011). Many studies have focused on elucidating the underlying mechanisms of the origin and maintenance of genetic variation in populations of scleractinian corals as they provide much of the habitat framework and structural complexity of reefs (e.g., Baums, Miller & Hellberg, 2005; Baums, 2008; Davies, Treml & Matz, 2015).

For long-live sessile organisms, such as reef-building corals, patterns of genetic diversity at both local and global scales are highly governed by the dispersal of sexual larvae (Baird, Guest & Willis, 2009; Harrison, 2011). Molecular studies have uncovered a wide range of dispersal patterns in scleractinian corals, from populations primarily sustained by self-recruitment (Gilmour, Smith & Brinkman, 2009; Mokhtar-Jamaï et al., 2013) through ecologically significant gene flow and connectivity among their populations (Van der Ven et al., 2016; Lukoschek, Riginos & Van Oppen, 2016). Furthermore, the degree of genetic variation in partially clonal reef organisms is heavily influenced by the relative contribution from sexual and asexual reproduction for local population maintenance (e.g., Baums, Miller & Hellberg, 2006; Pinzón et al., 2012; Adjeroud et al., 2014). While our understanding of population genetics in scleractinian corals has improved considerably over the last decade, such information remains unavailable for *Millepora* hydrocorals (‘fire corals’).

*Millepora* hydrocorals are an important component of reef communities worldwide where they, similar to scleractinian corals, significantly contribute to reef accretion (Nagelkerken & Nagelkerken, 2004; Lewis, 2006). Although fire corals compete with other reef-building taxa (Wahle, 1980; Dubé, Boissin & Planes, 2016), they also favour coral survival during *Acanthaster* outbreaks, highlighting their key ecological role in reef resilience (Kayal & Kayal, 2016). Despite their major importance for reef conservation, fire corals have been relatively understudied and not much is known with respect to their genetic diversity, population structure or life history (e.g., reproductive strategies). Few studies have shown that *Millepora* species can colonise a wide range of reef environments via both sexual propagules (Lewis, 2006; Bourmaud et al., 2013) and asexual reproduction through fragmentation (Edmunds, 1999; Lewis, 2006). While they are sessile and have limited tolerance to environmental changes (Lewis, 2006), species of Milleporidae have a wide distribution range, i.e., circumtropical (Boschma, 1956). Fire corals are also known for their extensive morphological variability, which has caused problems in resolving their systematics (Boschma, 1948). There is currently no agreement regarding the number of *Millepora* species and no phylogenetic study investigating the genetic relationships among them (but see Ruiz-Ramos, Weil & Schizas, 2014 for a species complex in the Caribbean). Although microsatellite loci have been identified in *Millepora alcicornis* (Ruiz-Ramos & Baums, 2014), there was a lack of highly variable genetic markers for this genus until very recently (but see Heckenhauer et al., 2014). The development of new molecular markers for *Millepora* species will increase our knowledge on the genetic diversity of a conspicuous reef-building organism across its geographic range. These microsatellite markers will enable further studies on the biological, ecological and evolutionary processes underlying the population persistence of *Millepora* hydrocorals.

Microsatellites, also known as simple sequence repeats (SSRs) or short tandem repeats (STRs), have emerged as one of the most powerful genetic markers in population and evolutionary genetics (Selkoe & Toonen, 2006). Improvements in next generation sequencing techniques have provided new opportunities for microsatellite isolation in non-model organisms (i.e., with no genetic information available) (Zhang et al., 2011), with a good representativeness of loci across the genome (Martin et al., 2010). Because microsatellites are codominant (Estoup et al., 1993), highly polymorphic (Schlötterer, 2000) and transferable among closely related species (Cheng et al., 2012), they are commonly used for a remarkable array of applications, such as inferring genetic diversity (Silva & Gardner, 2015; Nakajima et al., 2016) and population structure patterns (Noreen, Harrison & Van Oppen, 2009; Boissin et al., 2016), evaluating reproductive strategies (Baums et al., 2014; Ardehed et al., 2015) and parentage screening (Mourier & Planes, 2013; Warner, Willis & Van Oppen, 2016). Cross-species transferability has been successful in many species (Barbará et al., 2007; Reid, Hoareau & Bloomer, 2012; Maduna et al., 2014; Pirog et al., 2016) allowing for genetic studies in closely related species. However, the few studies that have investigated the efficiency of cross-species transferability of microsatellite loci have demonstrated a negative correlation between the genetic distance and the amplification success (Carreras-Carbonell, Macpherson & Pascual, 2008; Hendrix et al., 2010; Moodley et al., 2015). This constraint can hamper accurate comparisons of genetic diversity among more distantly related species.

Here, we used 454 GS-FLX sequencing technology to develop an additional set of *de novo* microsatellite markers for *Millepora platyphylla* to first assess its genetic diversity on Moorea’s reefs in French Polynesia. Secondly, we tested these new microsatellite loci for cross-species amplification in five other *Millepora* species: the branching *Millepora intricata, Millepora dichotoma* and *Millepora tenera,* the plate-like species *Millepora complanata* and the encrusting *Millepora exaesa* (Boschma, 1948). Lastly, genetic distances based on the 16S mitochondrial gene were estimated among these species and *M. platyphylla* to identify the transferability success of these newly developed microsatellites across the Milleporidae.

## Materials & Methods

### Preparation of genomic DNA for 454 sequencing

The calcification processes (Stanley, 2006) and metabolic pathways (Trench, 1979) of calcareous hydrozoans are supported by a symbiotic association with protozoan dinoflagellate algae of the genus *Symbiodinium.* To design species-specific markers, genomic DNA of *Symbiodinium* was removed from the animal tissue using a succession of extraction techniques. Candidate microsatellite repeats were isolated from a pool of 14 partially bleached fragments of *M. platyphylla* collected *in situ* on Moorea’s reefs to minimise the quantity of *Symbiodinium* in their tissues. Further mechanic (centrifugation) and genetic (positive and negative controls in PCR) techniques were applied to ensure microsatellites belonged to the animal only (see below). Fragments were homogenised in 1,000 µL of CHAOS buffer (4 M guanidine thiocyanate; 0.5% N-lauroyl sarcosine; 25 nM Tris–HCl pH 8; 0.1 M 2-mercaptoethanol) modified from Fukami et al. (2004). Samples were incubated at 60 °C for 2 h while rotating and then centrifuged at 1,500 rpms for 30 s to precipitate symbiont algae expelled from host cells. A total of 20 µL of the aqueous phase was examined under microscope to confirm the absence of *Symbiodinium*. Further potential contamination was tested by running microsatellites on pure cultures of zooxanthellae DNA (see below). 350 µL of CHAOS solution containing animal tissues was transferred to a new vial and 350 µL PEB (protein extraction buffer) was added (100 mM Tris pH 8; 10 mM ethylenediaminetetraacetic acid (EDTA); 0.1% Sodium dodecyl sulfate (SDS)). DNA was purified with phenol/chloroform (24:1) and precipitated with isopropanol as described by Mieog et al. (2009). Samples from the 14 colonies were pooled together to increase detection of polymorphism. A total of 1 µg of genomic DNA was sent to GenoScreen platform (Lille, France) for the development of the microsatellite library using 454 GS-FLX Titanium reagents as described in Malausa et al. (2011). Briefly, total DNA was mechanically fragmented and enriched for TG, TC, AAC, AAG, AGG, ACG, ACAT and ACTC repeat motifs. Enriched fragments were subsequently amplified and PCR products were purified and quantified. GS-FLX libraries were then carried out following manufacturer’s protocols and sequenced on a GS-FLX PTP. The Quality Detection Device (QDD) pipeline (Meglécz et al., 2010) was used to analyse the 454 sequences and to design primers for amplification of the detected microsatellite motifs. Primer pairs were then selected depending on the motif (di-, tri-, tetranucleotide), the number of repeats (≥5) and the product size (≥100 bp) and tested on agarose gels for amplification.

**Table 1:** Characterisation of *de novo* microsatellite loci and genetic diversity in the target species *Millepora platyphylla* collected in Moorea, French Polynesia.

Locus name	Primer Sequence 5′–3′	Dye	MP	Motif	T_A_ (°C)	GenBank accession	N	Size (bp)	Null	LD	Na	*H*_*o*_	*H*_*e*_	*F*_IS_
Mill07	F: TAGTACATCGGGCATGAGCA	6-FAM	3	(CA)_16_	57	KX670763	50	92–144	–	–	13	0.760	0.855	**0.121**^∗^
	R: GTACTCTACGGCGTGTGCGT													
Mill27	R: CTTTCGTTTCCGATCATTCC	VIC	3	(TG)_10_	57	KX670764	50	136–148	–	0.044	5	0.600	0.636	0.067
	R: TGCCAGAACTAAGTTATCACAGC													
Mill30	F: AGTTGGCTCTGAGTGCGAGT	NED	2	(TG)_11_	57	KX670765	50	203–211	–	0.025	4	0.680	0.648	−0.039
	R: CCTCGGTTTATGGCTGAGAT													
Mill47	F: AAGCGTGTAATGCACTCAAAGA	NED	2	(GA)_8_	57	KX670766	50	118–162	0.101	0.057	10	0.600	0.766	**0.227**^∗∗^
	R: AACAGAAGTCGAACTGAGTCAAAA													
Mill52	F: CCCTGAGGCATCGAAATATAA	6-FAM	1	(AC)_9_	60	KX670767	50	94–98	–	–	2	0.420	0.412	−0.010
	R: TGCAATTGATGGTATTTGCATT													
Mill56	F: TCTGCAGATTTTGCATCTCG	PET	1	(AGA)_6_	60	KX670768	50	194	–	–	1	0	0	–
	R: TAGCAACAATGCTTCGCTGA													
Mill61	F: AAATGAACTCGCCCAAAAGA	PET	4	(CAA)_7_	57	KX670769	50	163–166	–	0.048	2	0.480	0.467	0.044
	R: ACACTGTCGATTGTGTTCCAA													
Mill67	F: TTGCGAGTTTACTTACCAGGC	VIC	1	(TAGA)_6_	60	KX670770	50	259–359	0.144	0.039	11	0.420	0.588	**0.294**^∗∗^
	R: TGAAGCAAATGACAAGAGCAA													
Mill86	F: GCGCGAAAATAAATTAAGGAA	NED	4	(GTT)_5_	57	KX670771	50	106	–	–	1	0	0	–
	R: TCCAATCTGAATTCCACCCT													
Mill91	F: CACTTTCGCCATTGTTGCTA	PET	4	(CAA)_6_	57	KX670772	50	116	–	–	1	0	0	–
	R: AACGGAATTCGAATCATTGC													
Mill93	F: TGAAATTTTCCAGTGACATCAAA	6-FAM	2	(TGT)_7_	57	KX670773	50	91–100	–	0.055	3	0.260	0.339	0.243
	R: GCTAATTATGAAATAGCAACTCCTAAA													
Mill94	F: GCATAAAGAATAAAGCAGAGGCA	6-FAM	3	(GAA)_7_	57	KX670774	50	131–140	–	0.016	2	0.480	0.461	−0.032
	R: CAATTGTGGGGAAGTTCGTT													
Mill95	F: TCCATAGCTTCTGCCTCCTC	6-FAM	1	(TTG)_7_	60	KX670775	50	123–138	–	0.022	3	0.320	0.304	−0.042
	R: GCTGATGATGCTGTCGAAGA													
Mill101	F: AGTCCTTCAATTGGTGGGTG	PET	2	(CAA)_6_	57	KX670776	50	132–135	–	–	2	0.640	0.493	−0.289
	R: GAGATGATGACTGAGCAGCAG													
Mill103	F: TTAAAGCCAGAGACAGAGAGACA	VIC	3	(AG)_7_	57	KX670777	50	94–100	–	0.017	4	0.700	0.621	−0.117
	R: ATCAACAGTTTCCCCTGTGC													

**Notes.**

MP multiplex panel in which each locus was included T_A_ primer temperature annealing N number of individuals with reliable amplification Null proportion of null alleles LD proportion of allele comparisons showing significant linkage disequilibrium (*P* < 0.05) Na number of alleles *H*_*o*_ observed heterozygosity *H*_*e*_ expected heterozygosity *F*_IS_ inbreeding coefficient

Significant values of *F*_IS_ are indicated by bold values with ^∗^*P* < 0.05 and ^∗∗^*P* < 0.001.

### Microsatellite discovery and primer testing

A panel of 16 *M. platyphylla* colonies was used to optimise PCR amplification and identify polymorphic loci. Small fragments of tissue-covered skeleton (<2cm^3^) were incubated at 55 °C for 1 h in 450 µL of digest buffer with proteinase K (QIAGEN, Hilden, Germany). Genomic DNA was extracted using a QIAxtractor automated genomic DNA extraction instrument, according to manufacturer’s instructions. PCRs were performed in a final volume of 10 µL including 5 µL Type-it Multiplex PCR Master Mix (1×) (QIAGEN, Hilden, Germany), 3 µL RNase-free water, 1 µL primers (2 µM for both forward and reverse primers diluted in TE buffer) and 1 µL of template (10–50 ng/µL). The PCR program included an initial denaturing step of 5 min at 95 °C, followed by 40 cycles of 30 s at 95 °C, 90 s at optimal temperature (57–60 °C) depending on the microsatellite locus (see Table 1), and 30 s at 72 °C, followed by a final 30 min elongation step at 60 °C. The PCR products were electrophoresed on 2% agarose gels. For loci with high-quality and consistent amplification, the PCR was repeated on DNA template isolated from *Symbiodinium* strains (clade A to F identified based on 23S chloroplast rDNA, Table S1) to identify coral specific loci and to exclude putative *Symbiodinium* specific loci. *Symbiodinium* strains were provided by the BURR laboratory at Buffalo, US (BURR; http://www.nsm.buffalo.edu/Bio/burr/). For the loci that are specific to *Millepora*, the forward primer was fluorescently labelled with the G5 dye set including 6-FAM, VIC, NED and PET (Applied Biosystems, Foster City, CA). Amplified fragments were visualised on an Applied Biosystems 3730 Sequencer using a GeneScan 500 LIZ ladder.

### Sampling, genotyping and cross-species amplification

The optimised loci were genotyped in our target species in addition to five other *Millepora* species to test for their transferability. For the characterisation of newly developed microsatellites, small fragments (<2 cm^3^) from 50 *M. platyphylla* colonies were collected on the reefs of Moorea in French Polynesia (CITES - FR1298700028-E). For cross-species amplification transferability tests, samples were collected from various locations in the Indo-Pacific and the Caribbean for five other species of fire corals: 11 *M. intricata* in Papua New Guinea, 30 *M. dichotoma* in Europa Island (Mozambique Canal), 30 *M. tenera* and 14 *M. exaesa* both in Reunion Island and 30 *M. complanata* in Curaçao (Table S2). DNA from the 165 *Millepora* colonies was extracted as described above and optimised loci were combined in four multiplex panels according to their allele size range and primer annealing temperature (see MP in Table 1). PCRs (10 µL) were performed with 2 µM of labelled forward primer and reverse primer with the same amplification conditions described above. PCR products were sent to GenoScreen (Lille, France) for fragment analysis and were visualised using an Applied Biosystems 3730 Sequencer. An internal size ladder (GeneScan 500 LIZ, Applied Biosystems) was used for accurate sizing and alleles were scored and checked manually using GENEMAPPER v.4.0 (Applied Biosystems, Foster City, CA). Samples that were ambiguous in scoring were re-amplified and re-scored. All peak profiles that were faint or ambiguous (i.e., multiple peaks) were considered as missing data.

### Phylogenetic analyses

Additionally, a 461 bp portion of the mitochondrial 16S gene was amplified for 30 specimens (five colonies per species) and used to estimate the genetic distances among the six *Millepora* species. The PCR amplifications were performed using the primers 16S-SHA and 16S-SHB (Cunningham & Buss, 1993) in 20 µL reactions containing: 1.5 mM of MgCl_2_, 0.2 mM of each dNTP, 1× final concentration of buffer, 0.5 µM of each primer, 0.25 unit of Red Hot Taq polymerase, 2 µL of DNA template (80–100 ng/µL) and sterilised water up to 20 µL. The cycling parameters were as follows: an initial denaturation step of 5 min at 94 °C, followed by 35 cycles of 1 min at 94 °C, 1 min at 50 °C, 1.5 min at 72 °C and a final elongation step of 5 min at 72 °C. Sequencing of the PCR products was performed by GenoScreen (Lille, France).

### Data analyses

Control for the presence of null alleles, scoring errors and large allele dropout were performed with MICROCHECKER v.3.7 (Van Oosterhout et al., 2004). To assess the discriminative power of the microsatellite markers, the genotype probability (GP) was estimated for each locus and for a combination of all loci using GENALEX v.6.5 (Peakall & Smouse, 2006). Repeated multilocus genotypes (MLGs) were also identified in GENALEX and were considered as clone mates at GP < 0.001. The probability of identity, *P*_(ID)_, was computed to evaluate the power of our microsatellites to accurately distinguish closely related genotypes from those produced by asexual reproduction (Waits, Luikart & Taberlet, 2001). Population genetic analyses were then performed after the removal of all clonal replicates.

Indices of genetic diversity were estimated for each species in all locations using GENALEX, including Na, the total number of alleles per locus, observed (*H*_*o*_) and expected (*He*) heterozygosity (Weir & Cockerham, 1984). The inbreeding coefficient *F*_IS_ and linkage disequilibrium were estimated using GENETIX v.4.02 (Belkhir et al., 1996), applying a permutation procedure (1,000 permutations) to assess statistical significance. GENETIX was also used to estimate genetic distance among populations of *M. platyphylla* and the other *Millepora* species with the microsatellite dataset using the *θ* estimator of *F*_ST_ (Weir & Cockerham, 1984) based on a permutation procedure (1,000 permutations). Genetic *p*-distances among species at the mtDNA 16S gene were calculated in Mega v.6 (Tamura et al., 2013). In addition to the *p*-distance, we also computed other genetic distances (i.e., Kimura-2-Parameters, Tamura & Nei and Maximum composite Likelihood, all available in the software Mega v.6) and found similar species rank among all measures tested. We also examined the cross-species amplification success of the new microsatellite markers by plotting the genetic diversity (*Ho*) and the proportions of missing data (non amplified loci after 3× repeat PCR, and this at different annealing temperatures) in each species against genetic distance (16S) and relationships were tested using Pearson’s correlation coefficient.

## Results

### Development of *de novo* microsatellites in *Millepora platyphylla*

Sequencing of the microsatellite-enriched library from 14 partially bleached fragments of *M. platyphylla* yielded 78,784 reads. The Quality Detection Device (QDD) for bioinformatic filtering resulted in a final set of 5,976 sequences containing microsatellite motifs. For the characterisation of new microsatellites, 127 primer pairs (out of the 186 resulting from the QDD filtering, 68.3%) were tested in 16 individuals of *M. platyphylla* collected on Moorea’s reefs. Fifteen loci showed clear amplification profiles and no *Symbiodinium* specific locus was identified, proving the efficiency of the DNA extraction technique. For the 50 *M. platyphylla* colonies collected on Moorea’s reefs, twelve loci were polymorphic (from 2 to 13 alleles) and three additional monomorphic loci were retained for further cross-species transferability tests (Table 1). Contig sequences containing the microsatellites identified in this study are available in GenBank (KX670763 –KX670777, Table 1).

Significant linkage disequilibrium was identified and distributed among all microsatellite loci in *M. platyphylla.* 9.1% of the pairwise locus combinations showed a significant probability of linkage disequilibrium at *P* < 0.05 (Table 2). The presence of null alleles was detected at Mill47 and Mill67 with frequencies of null alleles at both loci estimated at 10.1% and 14.4%, respectively. These two loci were removed from our dataset for further genetic analyses, although there was no evidence of scoring error or large allele dropout for any locus. Given the low *P*_(ID)_ value estimated (1.3E−6), our panel of microsatellites had a high power to distinguish colonies that were clonal replicates. For the ten polymorphic loci showing no evidence of null alleles, the mean number of alleles (Na) per locus was 3.462 and the observed heterozygosity (*Ho*) was 0.411 (Table 2). Only three loci out of fifteen showed significant deficiency in heterozygotes compared to HWE and only one of them showed no evidence of null alleles (Mill07, *F*_IS_: 0.121, Table 1).

**Table 2:** Summary of genetic distances (GD) based on the 16S gene between the target species and other *Millepora* species together with indices indicating microsatellite transferability and genetic diversity.

Species	Locality	N	MLG	Clonal MLG	*P*_(ID)_	GD	Amp (%)	Pol (%)	Null (%)	LD (%)	Na	*H*_*o*_
*M. platyphylla*	Moorea	50	50	–	1.3E−6	–	100	80.0	13.3	9.1	3.462	0.411
*M. intricata*	Papua	11	10	1	1.1E−6	0.048	73.3	60.0	–	12.1	3.909	0.405
*M. dichotoma*	Europa	30	24	4	4.1E−7	0.049	86.7	60.0	7.7	10.3	3.417	0.323
*M. tenera*	Reunion	30	24	6	3.1E−7	0.049	80.0	73.3	58.3	23.0	4.833	0.439
*M. complanata*	Curaçao	30	30	–	1.3E−6	0.130	53.3	46.7	75.0	10.2	4.000	0.250
*M. exaesa*	Reunion	14	14	–	3.9E−6	0.149	60.0	53.3	11.1	17.6	3.625	0.496

**Notes.**

N sample size MLG number of multilocus genotypes Clonal MLG number of multilocus genotypes with clonal replicates *P*_(ID)_ Probability of Identity Amp percentage of loci amplified Pol percentage of polymorphic loci Null percentage of loci showing evidence of null alleles LD percentage of allele comparisons showing significant linkage disequilibrium (*P* < 0.05) Na mean number of alleles *H*_*o*_ mean observed heterozygosity

Na and *H*_*o*_ were estimated based on loci showing no evidence of null alleles and clonal replicates were removed from our dataset for these measures of genetic diversity.

**Table 3:** Nuclear (*F*_ST_) and mitochondrial (*p*-distance) genetic distances among *Millepora* species. Values above the diagonal are the *F*_ST_ calculated on the microsatellite dataset, values with *P* < 0.001 are in bold and the remaining values are NS. Values below the diagonal are genetic distances (*p*-distance) based on the mitochondrial 16S gene.

	*M. platyphylla*	*M. intricata*	*M. dichotoma*	*M. tenera*	*M. exaesa*
*M. platyphylla*		**0.343**	**0.373**	**0.339**	**0.167**
*M. intricata*	0.048		0.031	0.065	**0.181**
*M. dichotoma*	0.049	0.051		0.062	**0.221**
*M. tenera*	0.049	0.051	0.000		**0.293**
*M. exaesa*	0.149	0.143	0.150	0.150	

### Cross-species amplification in Milleporidae

**Figure 1:**
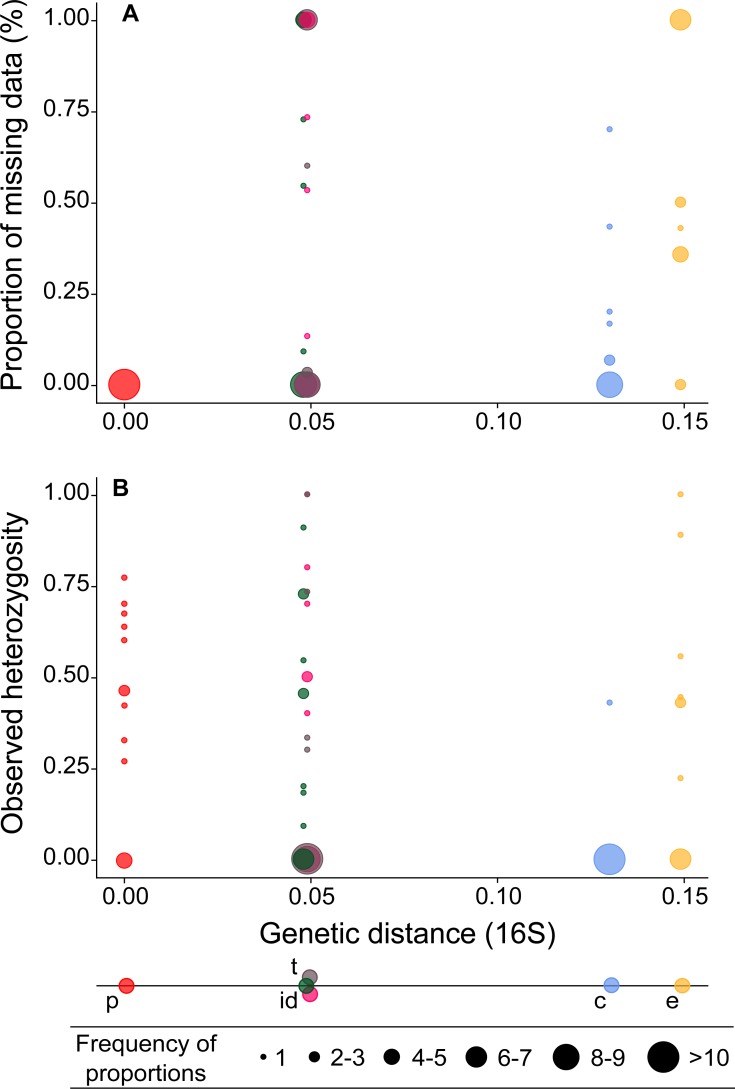
Proportion of missing data (A) and observed heterozygosity (B) per microsatellite locus (circles) in five *Millepora* species plotted against genetic distances (16S gene) from the target species. Target species *Millepora platyphylla* (p, red) and non-target species; *Millepora intricata* (i, green), *Millepora dichotoma* (d, pink), *Millepora tenera* (t, purple), *Millepora complanata* (c, blue) and *Millepora exaesa* (e, yellow).

Assessment of the mtDNA genetic distances (GD) within the *Millepora* genus revealed that branching species, i.e., *M. intricata, M. dichotoma* and *M. tenera*, were more closely related (0.048–0.049) to our target species, with haplotypes shared between *M. dichotoma* and *M. tenera* (Table 3 and see Appendix S1 for the haplotype network). The plate-like *M. complanata* (0.130) and encrusting *M. exaesa* (0.149) were more genetically distant from *M. platyphylla*. The mean amplification success for cross-species amplification was 70.7% (∼11 loci out of 15) and the mean polymorphism was 58.7% (∼9 loci out of 15). Cross-species amplification decreased significantly with mtDNA genetic distance (*r* =  − 0.931, *P* = 0.007), with a reduced amplification success in the most divergent species, i.e., *M. complanata* (53.3%) and *M. exaesa* (60.0%), and higher for *M. intricata* (73.3%), *M. tenera* (80.0%) and *M. dichotoma* (86.7%) (Table 2). Cross-species amplification also revealed a significant decrease of polymorphism with increasing mtDNA distance (*r* =  − 0.857, *P* = 0.029), with lower percentages of polymorphic loci for non-target species (≤73.3%, Table 2). No relationship was found between the percentage of loci showing evidence of null alleles and genetic distance (*r* = 0.331, *P* = 0.521). The highest percentage was recorded for *M. complanata* (75.0%), while lowest for *M. intricata* (0%). The proportion of missing data per locus increased significantly with increasing level of genetic distance (Fig. 1A, *r* = 0.214, *P* = 0.044).

Clonal replicates were found in the three branching species: 1 clonal MLG in *M. intricata,* 4 in *M. dichotoma* and 6 in *M. tenera* (Table 2). The mean observed heterozygosity per locus was highly variable in all species, although more limited in *M. tenera* and *M. complanata* due to high proportions of null alleles in both species (Fig. 1B and Table S3). No significant correlation was found between the genetic diversity and mtDNA genetic distance (*r* =  − 0.175, *P* = 0.101). The mean observed heterozygosity was slightly reduced for *M. complanata* (0.250) compared to other species (0.323 for *M. dichotoma* ⩽ *Ho* ⩽ 0.496 for *M. exaesa*) (Table 2). However, *Ho* estimate in *M. complanata* was based on only one microsatellite locus (Mill 103, Fig. 1B). For the four other non-target species, 2 loci out of 15 showed significant deficiencies in heterozygotes compared to HWE in *M. dichotoma* (Mill07 and Mill67, *F*_IS_: 1.000) and another one in *M. intricata* (Mill101, *F*_IS_: 0.500) (Table S3).

The transferability of microsatellites in the Milleporidae also revealed strong genetic differentiation among some species (Table 3 and see Appendix S2 for the Bayesian clustering analysis). No significant genetic differentiation was observed for the closely related branching species (i.e., *M. intricata, M. dichotoma* and *M. tenera*). For all comparisons involving our target species *M. platyphylla,* the lowest value of *F*_ST_(≤0.167) was recorded for the most divergent species *M. exaesa.* No relationship (*r* = 0.150, *P* = 0.679) was found between the nuclear (*F*_ST_ from microsatellite data) and mitochondrial (*p*-distance from 16S) genetic distances.

## Discussion

### Microsatellites’ development and transferability in Milleporidae

To date, there is no study assessing the genetic diversity and population structure of fire coral species. This gap is mostly due to the lack of highly variable genetic markers in the genus until very recently, whereas microsatellite loci have been identified in the Caribbean species *Millepora alcicornis* (Ruiz-Ramos & Baums, 2014). Heckenhauer et al. (2014) have succeeded in developing eleven microsatellite markers for *M. dichotoma* from the Great Barrier Reef and showed that their transferability was successful between geographic regions (Red Sea) and the species *M. platyphylla*. Their study has shown that eight of the eleven microsatellite markers (72.7%) were transferable to *M. platyphylla* which is less to what we had in the present study (i.e., 86.7% between *M. dichotoma* and *M. platyphylla*). Six of their loci had only 2 alleles for *M. platyphylla*, which is not informative enough depending on the analyses performed (e.g., parentage analyses). Furthermore, most of the microsatellite markers developed by Heckenhauer et al. (2014) were characterised by significant deficiencies in heterozygotes, whereas only two of our loci showed such HWE disequilibrium in both of these species. Depending on the target species, a combination of markers from the two studies would thus seem a good strategy for population genetic approaches in *Millepora* hydrocorals.

Our cross-amplification tests show a higher cross-taxon transferability success (73.3–86.7%) for a genetic distance below 5% from our target species (i.e., *M. intricata, M. dichotoma* and *M. tenera*) and a reduced transferability above this level (≤60% for *M*. *complanata* and *M. exaesa*). Overall, our results show a high probability to amplify a microsatellite locus within the genus *Millepora*, where 71% of the loci were successfully amplified in the five non-target species. This value is slightly lower to what was demonstrated for the Caribbean *Montastraea* species complex (scleractinian corals), i.e., ∼80% of amplification success in two other species within the same location (Davies et al., 2013). Our lower value, while still very high, is not surprising as we tested cross-amplification between six species of the genus *Millepora* (i.e., no species complex as for *Montastraea* spp), which were also collected throughout their entire geographic range. The non-amplification of some microsatellite loci in the non-target species is most likely due to specific mutations in the primer binding sites in *M. platyphylla,* i.e., null alleles (Paetkau & Strobeck, 1995). These loci, specific to Moorea’s population, may result from local evolutionary processes at this location, such as bottlenecks, expansions, life history traits, inbreeding and outbreeding (Keller & Waller, 2002; Leffler et al., 2012; Romiguier et al., 2014). Our cross-amplified loci show a high probability to be polymorphic in non-target species (58.7%), which is much higher to what is generally found in other taxa, such as fishes (∼25–30% in Barbará et al., 2007; Reid, Hoareau & Bloomer, 2012) and birds (∼20–50% in Dawson et al., 2010). Many other studies using cross-amplification have shown a significant decrease in the transferability success and polymorphism with evolutionary distance from the target species (Jan et al., 2012; Maduna et al., 2014; Moodley et al., 2015).

### Usefulness of cross-species amplification in Indo-Pacific Milleporidae

The level of genetic diversity is key for the persistence of a species in changing environments and represents a fundamental aspect of biodiversity (Romiguier et al., 2014). Quantifying the genetic diversity in natural populations and species is critical to address ecological and evolutionary questions (Nair, 2014), which requires the development of suitable molecular resources. In this study, our cross-species amplification approach for the development of new microsatellites shows no significant evidence of lower genetic diversity nor greater proportion of null alleles with increasing genetic distance from our target species, which is in contradiction with previous studies (Carreras-Carbonell, Macpherson & Pascual, 2008; Hendrix et al., 2010; Moodley et al., 2015). Our results also show that most of our microsatellite markers are not useful to estimate the genetic diversity in the Caribbean species *M. complanata* due to the high proportion of null alleles. Hence, this study reveals that the transferability of our microsatellites ensures comparable estimations of the genetic diversity among closely related *Millepora* species, although restricted to the Indo-Pacific region. Further investigations with other Caribbean species, such as *M. alcicornis,* are needed to test their transferability in this geographic region.

In this study, we also found that genetic distance from interspecific microsatellite data were not congruent with mtDNA distance among the studied species. It is not surprising as such highly variable markers would suffer from homoplasy as one look into higher taxon relationships, while microsatellites are well-known to be mostly useful for intra-specific studies (Selkoe & Toonen, 2006). Nonetheless, assessment of the population structure among closely related Indo-Pacific species revealed a clear genetic differentiation between the branching species and the plate-like *M. platyphylla*. Our panel of new microsatellite loci is therefore useful for species delineation and can help resolve the century-old species problem in Milleporids (Boschma, 1948).

### Patterns of genetic diversity and population structure in Milleporidae

The first evaluation of genetic diversity among species of *Millepora* across its geographic range in tropical reefs reveals moderate levels of heterozygosity and allelic richness. The lowest genetic diversity was found for the Caribbean species, *M. complanata,* likely resulting from the low proportion of polymorphic loci (46.7%) and the high proportion of loci showing evidence of null alleles (75.0%). Nonetheless, levels of genetic diversity estimated in this study are similar to what was described for many tropical scleractinian species (Baums, 2008; Shearer, Porto & Zubilaga, 2009) and to what is expected in populations of partially clonal organisms. In this study, linkage disequilibrium, relatively high levels of allelic and genetic diversity, and heterozygote deficiencies were estimated for the six studied hydrocoral species, as previously described in some scleractinian corals (Baums, 2008). Overall, these new microsatellites are suitable to infer genetic diversity and to evaluate reproductive strategies in the partially clonal fire corals.

## Conclusions

This study highlights the utility of cross-species amplification of microsatellites in assessing population genetics of the *Millepora* genus in the Indo-Pacific region. Surprisingly, this approach does not result in lowering genetic diversity (*Ho*) in non-target species, thus ensuring an unbiased estimation of genetic diversity among fire coral species. The development of microsatellites can be complex and difficult in many taxa, such as birds (Primmer et al., 1997) and plants (Lagercrantz, Ellegren & Adersson, 1993), due to biological constraints that can affect the abundance and motif repeats of microsatellites in the genome (Tóth, Gáspári & Jurka, 2000). A recent study has demonstrated high microsatellite coverage in several species of cnidarians, including *Millepora alcicornis* (Ruiz-Ramos & Baums, 2014), indicating that there is no biological constraint for the development of microsatellite markers in this phylum. The availability of numerous microsatellite markers for reef-building *Millepora* species creates new opportunities for future research into the processes driving the complexity of their colonisation success on many Indo-Pacific reefs.

##  Supplemental Information

10.7717/peerj.2936/supp-1 Table S1 *Symbiodinium* strains used to identify coral specific loci See http://www.nsm.buffalo.edu/Bio/burr/ for more details on the *Symbiodinium* strains.

10.7717/peerj.2936/supp-2 Table S2 Sampling locations for populations of *Millepora*

10.7717/peerj.2936/supp-3 Table S3 Cross-species transferability of fifteen microsatellite loci isolated from *Millepora platyphylla* (Moorea) in five others species of *Millepora* *T*_*A*_, primer temperature annealing; Sp, Species; N, sample size; *N*_*amp*_, number of individuals with reliable amplification; Null, proportion of null alleles; LD, proportion of allele comparisons showing significant linkage disequilibrium (*P* < 0.05); Na, number of alleles; *H*_*o*_, observed heterozygosity; *H*_*e*_, expected heterozygosity; *F*_IS_, inbreeding coefficient. Significant values of *F*_IS_ are indicated by bold values with * *P* < 0.05, ** *P* < 0.01 and *** *P* < 0.001. Clonal replicates were removed from our dataset for the measures of genetic diversity.

10.7717/peerj.2936/supp-4 Appendix S1 Haplotype network of 16S sequences Each pie represents one 16S haplotype (with its area proportional to the number of individuals in which it was detected). The lengths of the grey lines connecting the 16S haplotypes are proportional to the number of mutations separating them with the number of mutations shown in red on each line. This haplotype network was reconstructed using the median joining algorithm (Bandelt, Forster & Rohl, 1999) in Network v5.0.0.0 (www.fluxus-engineering.com).
Bandelt HJ, Forster P, Rohl A. 1999. Median-joining networks for inferring intraspecific phylogenies. *Molecular Biology and Evolution* 16:37–48.

10.7717/peerj.2936/supp-5 Appendix S2 Bayesian clustering analysis Assignment analyses based on Bayesian clustering analysis using STRUCTURE (Pritchard, Stephens & Donnelly, 2000) for five of the six studied species: (1) *M. platyphylla,* (2) *M. exaesa*, (3) *M. intricata*, (4) *M. dichotoma* and (5) *M. tenera*. The *x*-axis shows species identification and y-axis shows the cluster membership ( *K* = 2). Initial STRUCTURE runs were used to determine the most likely number of clusters (K). Runs were performed with the default setting, a burn-in period of 50000, 50000 MCMC repeats and 10 iterations per K. The results were uploaded to STRUCTURE HARVESTER (Earl & vonHoldt, 2011) and the most likely K was retained for a second run in STRUCTURE with a burn-in period of 500000, 500000 MCMC repeats, 10 iterations and uniform prior setting.
Earl DA, vonHoldt BM. 2011. STRUCTURE HARVESTER: a website and program for visualizing STRUCTURE output and implementing the Evanno method. *Conservation Genetics Resources* 4:359–361. Pritchard JK, Stephens M, Donnelly P. 2000. Inference of population structure using multilocus genotype data. *Genetics* 155:945–959.

10.7717/peerj.2936/supp-6 Data S1 Microsatellite data for the six *Millepora* species — indicates no amplification.

## Additional Information and Declarations

### Field Study Permissions

The following information was supplied relating to field study approvals (i.e., approving body and any reference numbers): CITES - FR1298700028-E; field experiments were approved by the Presidency of French Polynesia (#0085).

### DNA Deposition

The following information was supplied regarding the deposition of DNA sequences: Genbank BankIt1941788 Seq_Mill1 KX670763
BankIt1941788 Seq_Mill2 KX670764
BankIt1941788 Seq_Mill3 KX670765
BankIt1941788 Seq_Mill4 KX670766
BankIt1941788 Seq_Mill5 KX670767
BankIt1941788 Seq_Mill6 KX670768
BankIt1941788 Seq_Mill7 KX670769
BankIt1941788 Seq_Mill8 KX670770
BankIt1941788 Seq_Mill9 KX670771
BankIt1941788 Seq_Mill10 KX670772
BankIt1941788 Seq_Mill11 KX670773
BankIt1941788 Seq_Mill12 KX670774
BankIt1941788 Seq_Mill13 KX670775
BankIt1941788 Seq_Mill14 KX670776
BankIt1941788 Seq_Mill15 KX670777.

### Data Availability

The following information was supplied regarding data availability: The raw data has been supplied as Data S1.
