# Supplementary material for: Genetic diversity and differentiation in reef-building Millepora species, as revealed by cross-species amplification of fifteen novel microsatellite loci
Source: PeerJ. 2017 Feb 23;5:e2936. doi: 10.7717/peerj.2936 (PMC5326544; doi:10.7717/peerj.2936)
Supplement: Table S1 — See http://www.nsm.buffalo.edu/Bio/burr/ for more details on the Symbiodinium strains. [file peerj-05-2936-s001.docx]

| **Culture ID** | **Host** | **Location** | **18S rDNA** | **Genotype**  **(23S chloroplast rDNA)** |
| --- | --- | --- | --- | --- |
|  |  |  |  |  |
|  |  |  |  |  |
| FLAP1 | *Aiptasia pallida* | Florida | A | A193 |
|  |  |  |  |  |
| Cass KB8 | *Cassiopea* sp. | Hawaii | A | A194 |
|  |  |  |  |  |
| Pe | *Porites evermanni* | Hawaii | B | B184 |
|  |  |  |  |  |
| FLAP2 | *Aiptasia pallida* | Florida | B | B184 |
|  |  |  |  |  |
| Mp | *Mastigia paupa* | Palau | C | C180 |
|  |  |  |  |  |
| A001 | *Acropora* sp*.* | Okinawa | D | D206 |
|  |  |  |  |  |
| A014 | *Porites australiensis* | Okinawa | D | D206 |
|  |  |  |  |  |
| CCMP421 | unknown | New Zealand | E | E202 |
|  |  |  |  |  |
| Pd | *Porites divaricata* | Florida | F | F178 |
|  |  |  |  |  |
| Sin | *Sinularia* sp. | Guam | F | F179 |
|  |  |  |  |  |
