# Supplementary material for: Genetic diversity and differentiation in reef-building Millepora species, as revealed by cross-species amplification of fifteen novel microsatellite loci
Source: PeerJ. 2017 Feb 23;5:e2936. doi: 10.7717/peerj.2936 (PMC5326544; doi:10.7717/peerj.2936)
Supplement: Table S2 [file peerj-05-2936-s002.docx]

|  |  |  |  |  |
| --- | --- | --- | --- | --- |
| Species | Locality | Latitude (D.d) | Longitude (D.d) | Year |
|  |  |  |  |  |
|  |  |  |  |  |
| *M. platyphylla* | Moorea | 17.4816 S | 149.8755 W | 2013 |
|  |  |  |  |  |
| *M. intricata* | Papua New Guinea | 4.9937 S | 146.3299 E | 2014 |
|  |  |  |  |  |
| *M. dichotoma* | Europa | 20.3472 S | 40.3667 E | 2013 |
|  |  |  |  |  |
| *M. tenera* | Reunion | 20.9029 S | 55.3537 E | 2009 |
|  |  |  |  |  |
| *M. complanata* | Curaçao | 12.1202 N | 68.9696 W | 2014 |
|  |  |  |  |  |
| *M. exaesa* | Reunion | 20.9029 S | 55.3537 E | 2009 |
|  |  |  |  |  |
