# Supplementary material for: Genetic diversity and differentiation in reef-building Millepora species, as revealed by cross-species amplification of fifteen novel microsatellite loci
Source: PeerJ. 2017 Feb 23;5:e2936. doi: 10.7717/peerj.2936 (PMC5326544; doi:10.7717/peerj.2936)
Supplement: Table S3 — TA, primer temperature annealing; Sp, Species; N, sample size; Namp, number of individuals with reliable amplification; Null, proportion of null alleles; LD, proportion of allele comparisons showing significant linkage disequilibrium (P < 0.05); Na, number of alleles; Ho, observed heterozygosity; He, expected heterozygosity; FIS, inbreeding coefficient. Significant values of FIS are indicated by bold values with * P < 0.05, ** P < 0.01 and *** P < 0.001. Clonal replicates were removed from our dataset for the measures of genetic diversity. [file peerj-05-2936-s003.docx]

| Locus name | T_A_ (°C) | Sp | N | N_amp_ | Size (bp) | Null | LD | Na | *H_O_* | *H_E_* | *F_IS_* |
| --- | --- | --- | --- | --- | --- | --- | --- | --- | --- | --- | --- |
| Mill07 | 57 | *M. intricata* | 11 | 0 | 0 | --- | --- | 0 | --- | --- | --- |
|  |  | *M. dichotoma* | 30 | 8 | 96–102 | --- | 0.119 | 4 | 0.000 | 0.688 | **1.000***** |
|  |  | *M. tenera* | 30 | 0 | 0 | --- | --- | 0 | --- | --- | --- |
|  |  | *M. complanata* | 30 | 9 | 92–106 | 0.237 | 0.077 | 9 | 0.444 | 0.839 | **0.515***** |
|  |  | *M. exaesa* | 14 | 0 | 0 | --- | --- | 0 | --- | --- | --- |
| Mill27 | 57 | *M. intricata* | 11 | 11 | 156–188 | --- | 0.026 | 15 | 0.900 | 0.920 | 0.052 |
|  |  | *M. dichotoma* | 30 | 30 | 150–190 | --- | 0.133 | 16 | 1.000 | 0.914 | –0.073 |
|  |  | *M. tenera* | 30 | 30 | 138–198 | --- | 0.158 | 15 | 1.000 | 0.901 | –0.089 |
|  |  | *M. complanata* | 30 | 0 | 0 | --- | --- | 0 | --- | --- | --- |
|  |  | *M. exaesa* | 14 | 0 | 0 | --- | --- | 0 | --- | --- | --- |
| Mill30 | 57 | *M. intricata* | 11 | 0 | 0 | --- | --- | 0 | --- | --- | --- |
|  |  | *M. dichotoma* | 30 | 0 | 0 | --- | --- | 0 | --- | --- | --- |
|  |  | *M. tenera* | 30 | 12 | 203–213 | 0.295 | 0.095 | 5 | 0.300 | 0.680 | **0.594***** |
|  |  | *M. complanata* | 30 | 19 | 205–211 | 0.368 | 0.035 | 4 | 0.118 | 0.642 | **0.910***** |
|  |  | *M. exaesa* | 14 | 9 | 203–209 | --- | 0.060 | 4 | 0.556 | 0.685 | 0.245 |
| Mill47 | 57 | *M. intricata* | 11 | 11 | 116–122 | --- | 0.045 | 4 | 0.700 | 0.715 | 0.018 |
|  |  | *M. dichotoma* | 30 | 26 | 116–128 | 0.374 | 0.219 | 5 | 0.150 | 0.779 | **0.816***** |
|  |  | *M. tenera* | 30 | 30 | 114–128 | 0.144 | 0.200 | 5 | 0.542 | 0.691 | 0.236 |
|  |  | *M. complanata* | 30 | 30 | 118 | --- | --- | 1 | 0 | 0 | --- |
|  |  | *M. exaesa* | 14 | 7 | 116–120 | --- | 0.078 | 3 | 0.429 | 0.602 | 0.357 |
| Mill52 | 60 | *M. intricata* | 11 | 10 | 94 | --- | --- | 1 | 0 | 0 | --- |
|  |  | *M. dichotoma* | 30 | 30 | 94 | --- | --- | 1 | 0 | 0 | --- |
|  |  | *M. tenera* | 30 | 30 | 94–96 | 0.189 | 0.089 | 2 | 0.083 | 0.219 | **0.632*** |
|  |  | *M. complanata* | 30 | 0 | 0 | --- | --- | 0 | --- | --- | --- |
|  |  | *M. exaesa* | 14 | 14 | 94–126 | --- | 0.034 | 4 | 0.429 | 0.612 | 0.333 |
| Mill56 | 60 | *M. intricata* | 11 | 11 | 194–197 | --- | 0.049 | 2 | 0.200 | 0.180 | –0.053 |
|  |  | *M. dichotoma* | 30 | 30 | 194–200 | --- | 0.194 | 3 | 0.458 | 0.378 | –0.193 |
|  |  | *M. tenera* | 30 | 30 | 194–197 | --- | 0.044 | 2 | 0.708 | 0.457 | –0.533 |
|  |  | *M. complanata* | 30 | 30 | 169–200 | 0.201 | 0.036 | 3 | 0.200 | 0.383 | **0.490**** |
|  |  | *M. exaesa* | 14 | 14 | 194 | --- | --- | 1 | 0 | 0 | --- |
| Mill61 | 57 | *M. intricata* | 11 | 0 | 0 | --- | --- | 0 | --- | --- | --- |
|  |  | *M. dichotoma* | 30 | 0 | 0 | --- | --- | 0 | --- | --- | --- |
|  |  | *M. tenera* | 30 | 0 | 0 | --- | --- | 0 | --- | --- | --- |
|  |  | *M. complanata* | 30 | 0 | 0 | --- | --- | 0 | --- | --- | --- |
|  |  | *M. exaesa* | 14 | 0 | 0 | --- | --- | 0 | --- | --- | --- |
| Mill67 | 60 | *M. intricata* | 11 | 5 | 249–265 | --- | 0.108 | 3 | 0.250 | 0.531 | 0.636 |
|  |  | *M. dichotoma* | 30 | 14 | 255–261 | --- | 0.114 | 2 | 0.000 | 0.153 | **1.000*** |
|  |  | *M. tenera* | 30 | 29 | 241–261 | 0.271 | 0.223 | 4 | 0.304 | 0.678 | **0.566***** |
|  |  | *M. complanata* | 30 | 0 | 0 | --- | --- | 0 | --- | --- | --- |
|  |  | *M. exaesa* | 14 | 0 | 0 | --- | --- | 0 | --- | --- | --- |
| Mill86 | 57 | *M. intricata* | 11 | 0 | 0 | --- | --- | 0 | --- | --- | --- |
|  |  | *M. dichotoma* | 30 | 30 | 97 | --- | --- | 1 | 0 | 0 | --- |
|  |  | *M. tenera* | 30 | 0 | 0 | --- | --- | 0 | --- | --- | --- |
|  |  | *M. complanata* | 30 | 0 | 0 | --- | --- | 0 | --- | --- | --- |
|  |  | *M. exaesa* | 14 | 0 | 0 | --- | --- | 0 | --- | --- | --- |
| Mill91 | 57 | *M. intricata* | 11 | 11 | 116 | --- | --- | 1 | 0 | 0 | --- |
|  |  | *M. dichotoma* | 30 | 30 | 116 | --- | --- | 1 | 0 | 0 | --- |
|  |  | *M. tenera* | 30 | 30 | 116 | --- | --- | 1 | 0 | 0 | --- |
|  |  | *M. complanata* | 30 | 25 | 101–194 | 0.410 | 0.078 | 7 | 0.08 | 0.7192 | **0.893***** |
|  |  | *M. exaesa* | 14 | 0 | 0 | --- | --- | 0 | --- | --- | --- |
| Mill93 | 57 | *M. intricata* | 11 | 11 | 91–103 | --- | 0.090 | 4 | 0.500 | 0.655 | 0.359 |
|  |  | *M. dichotoma* | 30 | 30 | 91–100 | --- | 0.248 | 3 | 0.458 | 0.499 | 0.103 |
|  |  | *M. tenera* | 30 | 29 | 94–97 | 0.291 | 0.255 | 2 | 0.174 | 0.454 | **0.630**** |
|  |  | *M. complanata* | 30 | 0 | 0 | --- | --- | 0 | --- | --- | --- |
|  |  | *M. exaesa* | 14 | 8 | 91–97 | --- | 0.033 | 3 | 1.000 | 0.555 | –0.061 |
| Mill94 | 57 | *M. intricata* | 11 | 11 | 122–140 | --- | 0.019 | 4 | 0.500 | 0.415 | –0.176 |
|  |  | *M. dichotoma* | 30 | 30 | 128–140 | --- | 0.173 | 4 | 0.750 | 0.635 | –0.161 |
|  |  | *M. tenera* | 30 | 30 | 128–137 | --- | 0.128 | 4 | 0.333 | 0.411 | 0.209 |
|  |  | *M. complanata* | 30 | 25 | 128–140 | 0.224 | 0.063 | 5 | 0.292 | 0.673 | **0.581***** |
|  |  | *M. exaesa* | 14 | 7 | 128–138 | 0.372 | 0.034 | 4 | 0.143 | 0.684 | **0.818*** |
| Mill95 | 60 | *M. intricata* | 11 | 11 | 123–126 | --- | 0.049 | 2 | 0.500 | 0.455 | 0.000 |
|  |  | *M. dichotoma* | 30 | 30 | 120–126 | --- | 0.240 | 3 | 0.667 | 0.622 | –0.051 |
|  |  | *M. tenera* | 30 | 30 | 123–126 | --- | 0.289 | 2 | 0.292 | 0.457 | 0.381 |
|  |  | *M. complanata* | 30 | 28 | 120–171 | 0.186 | 0.069 | 7 | 0.357 | 0.576 | **0.396***** |
|  |  | *M. exaesa* | 14 | 9 | 111–141 | --- | 0.016 | 7 | 0.889 | 0.796 | –0.058 |
| Mill101 | 57 | *M. intricata* | 11 | 11 | 132–138 | --- | --- | 3 | 0.100 | 0.185 | **0.500*** |
|  |  | *M. dichotoma* | 30 | 30 | 135 | --- | --- | 1 | 0 | 0 | --- |
|  |  | *M. tenera* | 30 | 30 | 132–138 | 0.214 | 0.083 | 3 | 0.125 | 0.291 | **0.584***** |
|  |  | *M. complanata* | 30 | 0 | 0 | --- | --- | 0 | --- | --- | --- |
|  |  | *M. exaesa* | 14 | 9 | 132–144 | --- | 0.086 | 4 | 0.444 | 0.451 | 0.072 |
| Mill103 | 57 | *M. intricata* | 11 | 11 | 94–104 | --- | 0.019 | 4 | 0.800 | 0.625 | –0.151 |
|  |  | *M. dichotoma* | 30 | 30 | 94–96 | --- | 0.250 | 2 | 0.542 | 0.492 | –0.079 |
|  |  | *M. tenera* | 30 | 29 | 94–96 | 0.389 | 0.489 | 2 | 0.043 | 0.496 | **0.916***** |
|  |  | *M. complanata* | 30 | 28 | 92–98 | --- | 0.486 | 4 | 0.429 | 0.580 | **0.278*** |
|  |  | *M. exaesa* | 14 | 9 | 94–98 | --- | 0.022 | 3 | 0.222 | 0.364 | 0.439 |
