# Supplementary figures and images for: Genetic diversity and differentiation in reef-building Millepora species, as revealed by cross-species amplification of fifteen novel microsatellite loci
Source: PeerJ. 2017 Feb 23;5:e2936. doi: 10.7717/peerj.2936 (PMC5326544; doi:10.7717/peerj.2936)

# Species

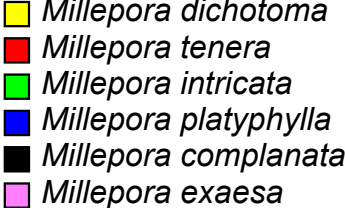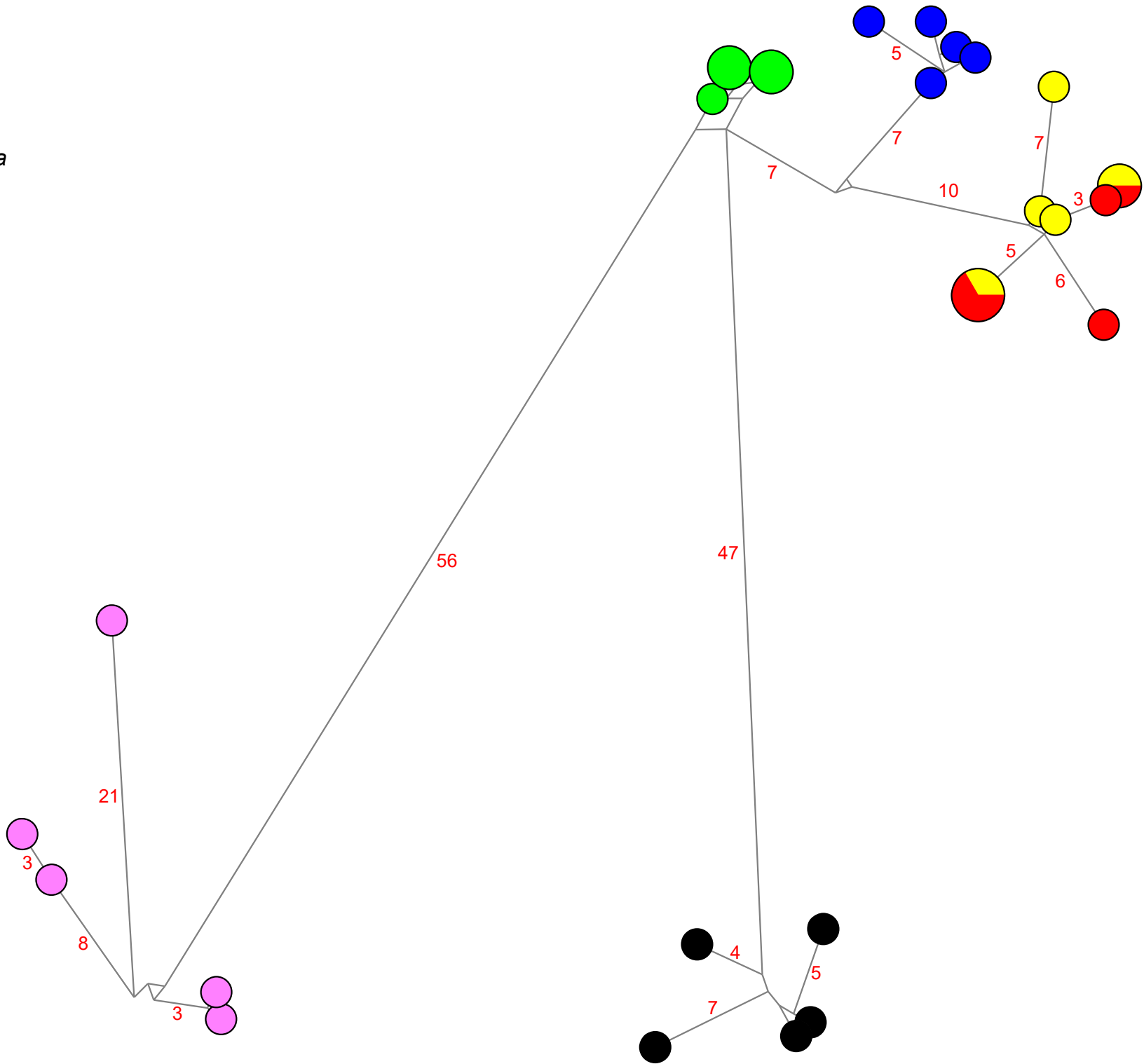

Supplement: Appendix S1 — Each pie represents one 16S haplotype (with its area proportional to the number of individuals in which it was detected). The lengths of the grey lines connecting the 16S haplotypes are proportional to the number of mutations separating them with the number of mutations shown in red on each line. This haplotype network was reconstructed using the median joining algorithm (Bandelt, Forster & Rohl, 1999) in Network v5.0.0.0 (www.fluxus-engineering.com). Bandelt HJ, Forster P, Rohl A. 1999. Median-joining networks for inferring intraspecific phylogenies. Molecular Biology and Evolution 16:37–48. [file peerj-05-2936-s004.pdf]

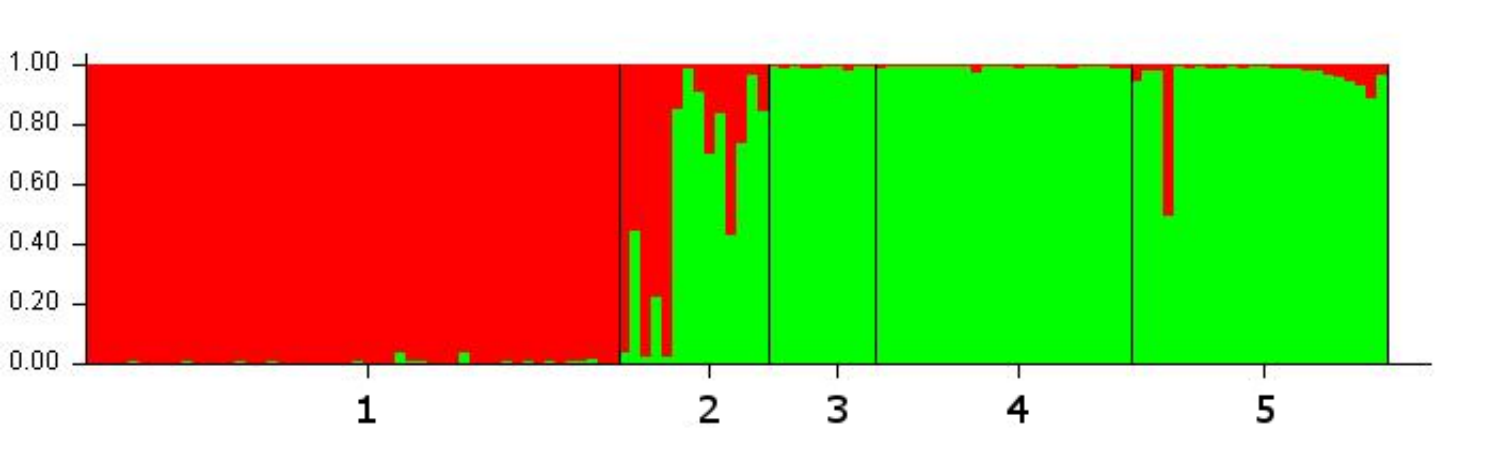

Supplement: Appendix S2 — Assignment analyses based on Bayesian clustering analysis using STRUCTURE (Pritchard, Stephens & Donnelly, 2000) for five of the six studied species: (1) M. platyphylla, (2) M. exaesa, (3) M. intricata, (4) M. dichotoma and (5) M. tenera. The x-axis shows species identification and y-axis shows the cluster membership ( K = 2). Initial STRUCTURE runs were used to determine the most likely number of clusters (K). Runs were performed with the default setting, a burn-in period of 50000, 50000 MCMC repeats and 10 iterations per K. The results were uploaded to STRUCTURE HARVESTER (Earl & vonHoldt, 2011) and the most likely K was retained for a second run in STRUCTURE with a burn-in period of 500000, 500000 MCMC repeats, 10 iterations and uniform prior setting. Earl DA, vonHoldt BM. 2011. STRUCTURE HARVESTER: a website and program for visualizing STRUCTURE output and implementing the Evanno method. Conservation Genetics Resources 4:359–361. Pritchard JK, Stephens M, Donnelly P. 2000. Inference of population structure using multilocus genotype data. Genetics 155:945–959. [file peerj-05-2936-s005.pdf]
